# Systematic screening on admission for SARS-CoV-2 to detect asymptomatic infections

**DOI:** 10.1186/s13756-021-00912-z

**Published:** 2021-02-27

**Authors:** Rahel N. Stadler, Laura Maurer, Lisandra Aguilar-Bultet, Fabian Franzeck, Chantal Ruchti, Richard Kühl, Andreas F. Widmer, Ruth Schindler, Roland Bingisser, Katharina M. Rentsch, Hans Pargger, Raoul Sutter, Luzius Steiner, Christoph Meier, Werner Kübler, Hans H. Hirsch, Adrian Egli, Manuel Battegay, Stefano Bassetti, Sarah Tschudin-Sutter

**Affiliations:** 1 Division of Infectious Diseases and Hospital Epidemiology, University Hospital Basel, University of Basel, Petersgraben 4, 4031 Basel, Switzerland; 2 Emergency Department, University Hospital Basel, University of Basel, Basel, Switzerland; 3 Laboratory Medicine, University Hospital Basel, University of Basel, Basel, Switzerland; 4 Intensive Care Unit, University Hospital Basel, University of Basel, Basel, Switzerland; 5 Anesthesiology, University Hospital Basel, University of Basel, Basel, Switzerland; 6 University Hospital Basel, University of Basel, Basel, Switzerland; 7 Clinical Virology, Laboratory Medicine, University Hospital Basel, Basel, Switzerland; 8 Transplantation and Clinical Virology, Department Biomedicine, University of Basel, Basel, Switzerland; 9 Clinical Microbiology, University Hospital Basel, University of Basel, Basel, Switzerland; 10 Internal Medicine, University Hospital Basel, University of Basel, Basel, Switzerland; 11 Department of Clinical Research, University Hospital Basel, University of Basel, Basel, Switzerland

**Keywords:** SARS-CoV-2, COVID-19, Asymptomatic carriers, Screening

## Abstract

The proportion of asymptomatic carriers of severe acute respiratory syndrome coronavirus 2 (SARS-CoV-2) remains elusive and the potential benefit of systematic screening during the SARS-CoV-2-pandemic is controversial. We investigated the proportion of asymptomatic inpatients who were identified by systematic screening for SARS-CoV-2 upon hospital admission. Our analysis revealed that systematic screening of asymptomatic inpatients detects a low total number of SARS-CoV-2 infections (0.1%), questioning the cost–benefit ratio of this intervention. Even when the population-wide prevalence was low, the proportion of asymptomatic carriers remained stable, supporting the need for universal infection prevention and control strategies to avoid onward transmission by undetected SARS-CoV-2-carriers during the pandemic.

## Background

Infections with the novel severe acute respiratory syndrome coronavirus 2 (SARS-CoV-2) display a wide range of symptoms ranging from fatal sepsis with pulmonary failure, classical symptoms of a viral respiratory tract infection, atypical symptoms, such as anosmia and ageusia and the lack of any detectable disease manifestation [1]. The proportion of asymptomatic SARS-CoV-2 infections remains elusive, with studies suggesting a range of 2–100% [2–5] depending on the studied population and the epidemiological setting. Asymptomatic carriers of SARS-CoV-2 may drive ongoing transmission of SARS-CoV-2, thus maintaining the pandemic [6, 7]. Although the secondary attack rate of asymptomatic carriers seems to be lower than that of symptomatic patients [3, 8], a relevant proportion of seemingly healthy but SARS-CoV-2 transmitting people may limit the impact of contact tracing and omission of subsequent spread significantly. Resources in terms of workforce, testing materials and personal protective equipment may be particularly restricted in the setting of a pandemic, so that they should be allocated to the most effective points of application. As the diagnostic yield of systematic screening to detect asymptomatic SARS-CoV-2-carriers on admission to an acute care facility is controversial, we investigated the proportion of asymptomatic SARS-CoV-2-infected inpatients.

## Methods

All adult patients admitted from 01.04.2020 to 14.06.2020 to the University Hospital Basel, a tertiary care center in Switzerland with more than 35,000 hospital admissions annually, were routinely tested for SARS-CoV-2 within 72 h of admission. Nasopharyngeal swabs were performed by a specially trained and dedicated team throughout the study period. An internally developed reverse transcription quantitative nucleic acid assay [9] and a commercial assay (E-gene; Roche, Rotkreuz, Switzerland) were used to detect SARS-CoV-2 RNA. Each patient screened during the study period was retrospectively classified as symptomatic or asymptomatic for coronavirus disease 2019 (COVID-19) at time of testing based on medical chart review. The classification criteria were the same as for the clinical consideration of COVID-19 and consistently applied during the screening period: acute pulmonary symptoms and/or fever ≥ 38.0 °C and/or sudden onset of anosmia or ageusia, and/or acute confusion or deterioration in the elderly, unless otherwise explained. The number of positive tested patients in the Canton Basel-Stadt, the main catchment area of the University Hospital Basel, was surveyed by and retrieved from the cantonal authorities [10] with most SARS-CoV-2 tests deriving from the triage- and test center of the University Hospital Basel [11]. Categorical variables were summarized as counts and proportions and continuous variables as medians and interquartile ranges. The Fisher’s exact test was used to assess differences between proportions. Approval by the local ethics committee was obtained (EKNZ-Number Req-2020-00808). We adhered to the Strengthening the Reporting of Observational Studies in Epidemiology (STROBE) guidelines [12].

## Results

**Fig. 1:**
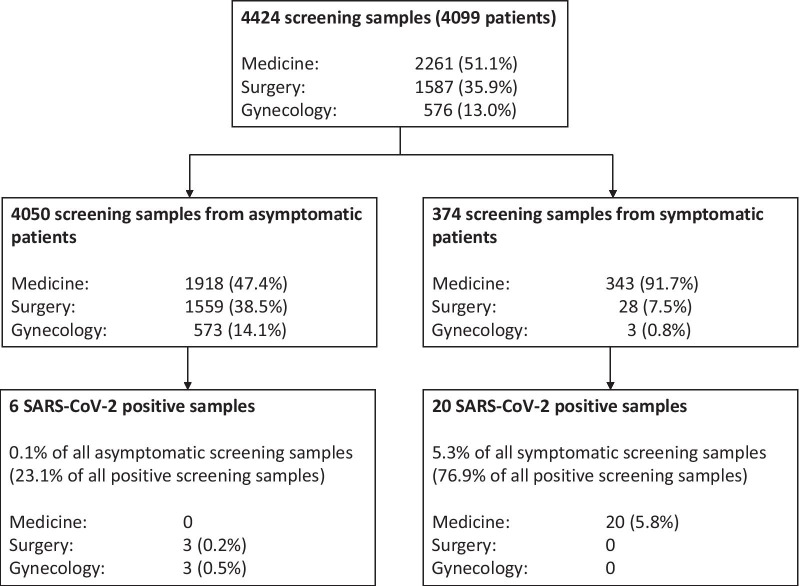
Results of SARS-CoV-2 screening samples from asymptomatic and symptomatic patients (overall and stratified by ward types)

During the study period, 4466 screening samples were collected from 4099 consecutive admitted patients. 49.9% of patients were female, the median age was 63 years (interquartile range 45–77 years). 2296 (51.4%), 1591 (35.6%), and 579 (13.0%) screenings were performed in the medical, surgical, and gynecology and obstetrics wards, respectively. Forty-two samples were excluded from further analyses, as they were collected for further follow-up (40 samples) or to screen contact patients (2 samples). 4050 (90.7%) screenings were classified as collected from asymptomatic patients at the time of testing. A total of 26 screenings (0.6%) tested positive for SARS-CoV-2: six of which were taken from asymptomatic patients (23.1% of all positive and 0.1% of all asymptomatic patients respectively) (Fig. 1). One out of six asymptomatic SARS-CoV-2-positive patients became symptomatic over the course of the hospitalization. However, symptoms could also be explained by the concomitant diagnosis of pyelonephritis. During the study period, the Canton Basel-Stadt reported 676 SARS-CoV-2-infections 44with an average population of 201′504 people (prevalence 0.3%) [10, 13]. The proportion of asymptomatic patients among all SARS-CoV-2-infected inpatients remained constant throughout the study period (21% (4/19) in April, 33% (2/6) in May, 0% (0/0) in June, p = 0.634), while the population-wide prevalence decreased.

## Discussion

Systematic screening of asymptomatic inpatients revealed a low total number of SARS-CoV-2 infections, yet a stable proportion of asymptomatic carriers over time.

This result is in line with other findings. Lavezzo et al. investigated the effects of lockdown and social distancing in the Italian town of Vo. They routinely tested over 70% of the community with nasopharyngeal swabs at the beginning of the lockdown in February 2020 and again, 2 weeks later, in March 2020. The prevalence of SARS-CoV-2 decreased from 2.6 to 1.2%. Symptoms in positive tested participants were systematically recorded. Notably, approximately 40% of people who tested positive were asymptomatic at both survey time-points [14]. The overall proportion of 23.1% of positive screenings in asymptomatic patients, identified in our study, was in the middle to lower range of reported asymptomatic infections in other studies: under 50% after direct contact with patients [3, 15] and in nursing home staff [16, 17], up to 75% in general population testing [14], up to 100% in health care workers [4], and 45% [18]–100% [5] in obstetric patients. In our sample, median age was 63 years. Higher age may correlate with the development of typical clinical symptoms of COVID-19 [19]. A recent meta-analysis found that in obstetric patients, 95% (45–100%) of SARS-CoV-2 infections were asymptomatic, whereas asymptomatic SARS-CoV-2 infection among nursing home residents occurred in 54% (42–65%) of patients [20] supporting a higher proportion of asymptomatic courses among younger age groups. High proportions of asymptomatic infections in both young and elderly people were found in an analysis of SARS-CoV-2 outbreaks in six care homes in Great Britain. The researchers swabbed every resident as well as the staff and followed them up for 14 days to classify individuals as being symptomatic, presymptomatic, postsymptomatic or asymptomatic throughout the study period. Among the elderly residents, nearly 40% tested positive, among the younger staff approximately 21%. In both groups, the proportion of asymptomatic individuals, was almost 64% at the time of testing. Over the course of the study, symptoms developed in 31.3% of asymptomatic residents and in 13.3% of the asymptomatic staff [21]. Extremely low asymptomatic carriage was predominantly reported early on in the pandemic [2, 22], which might have been due to testing being predominantly performed in symptomatic individuals.

Our study has some important limitations including the retrospective categorization of symptoms related to COVID-19 and its reliance on medical chart review. However, patients undergo a standardized evaluation during admission, improving the quality of such data. Furthermore, results are only generalizable to inpatients admitted to an acute care facility during a low endemicity setting or to younger patient populations, especially children. A higher total number of asymptomatic carriers of SARS-CoV-2 may have been identified, when screening in a higher endemicity setting and including younger patient populations.

## Conclusion

The high number needed to screen, to identify asymptomatic SARS-CoV-2 infected patients, questions the benefits of systematic on admission screening. The substantial proportion of asymptomatic SARS-CoV-2 infections, even in a low prevalence setting, emphasizes the need for universal infection and transmission control measures in health care institutions to prevent onward transmission by undetected SARS-CoV-2-carriers.

## Data Availability

The dataset generated and analyzed during the current study is available in the figshare repository, https://doi.org/10.6084/m9.figshare.13626026.v1. The relevant data of public datasets analyzed during the current study are available in the figshare repository, https://doi.org/10.6084/m9.figshare.13625984.v1 and https://doi.org/10.6084/m9.figshare.13626014.v1 [10, 13].

## Abbreviations

SARS-CoV-2: Severe acute respiratory syndrome coronavirus 2
RNA: Ribonucleic acid
COVID-19: Coronavirus disease 2019
EKNZ: Ethikkommission der Nordwest- und Zentralschweiz (Ethics Committee of Northwestern and Central Switzerland)
STROBE: Strengthening the Reporting of Observational Studies in Epidemiology Guidelines
